# A Retrospective Study of Lung Transplantation in Patients With Lymphangioleiomyomatosis: Challenges and Outcomes

**DOI:** 10.3389/fmed.2021.584826

**Published:** 2021-02-16

**Authors:** Ji Zhang, Dong Liu, Bingqing Yue, Le Ban, Min Zhou, Hongmei Wang, Jian Lv, Bo Wu, Zhenguo Zhai, Kai-Feng Xu, Wenhui Chen, Jingyu Chen

**Affiliations:** ^1^Wuxi Lung Transplant Center, Wuxi People's Hospital Affiliated to Nanjing Medical University, Wuxi, China; ^2^Department of Pulmonary and Critical Care Medicine, Center of Respiratory Medicine, China-Japan Friendship Hospital, Beijing, China; ^3^National Clinical Research Center for Respiratory Diseases, Peking University Health Science Center, Beijing, China; ^4^Department of Respiratory Medicine, Peking Union Medical College Hospital, Beijing, China; ^5^Department of Lung Transplantation, Center for Lung Transplantation, Center for Respiratory Diseases, China-Japan Friendship Hospital, Beijing, China

**Keywords:** lymphangioleiomyomatosis, lung transplantation, sirolimus, infection, anastomotic complication

## Abstract

**Background:** Lymphangioleiomyomatosis (LAM) is a rare systemic disease that generally leads to a progressive decline in pulmonary function. Experience, especially from the Asian population, including combined drug therapy before and after lung transplantation (LT) in LAM, is still limited. This study aimed to summarize the clinical data from patients with pulmonary LAM who underwent LT at centers in China.

**Methods:** A retrospective review of all patients with LAM undergoing LT at the two largest centers in China between 2010 and 2018 was conducted. Pre- and posttransplant data were assessed and analyzed.

**Results:** Overall, 25 patients with LAM underwent bilateral LT. The mean age was 35.0 ± 8.6 years at diagnosis and 36.8 ± 9.3 years at the time of transplant. Before LT, only six patients could complete pulmonary function test; the reachable mean forced expiratory volume in one second (FEV_1_) before LT was 15.9 ± 6.9%. Twenty-one patients (84%) had a recurrent pneumothorax, four (16.0%) of which required pleurodesis. Eight patients (32%) were treated with sirolimus pretransplant for 3.9 years (1–9 years). The average intra-surgery bleeding volume was 1,280 ± 730 ml in need of a transfusion of 1,316 ± 874 ml due to moderate-to-severe adhesion and pretransplant pleurodesis. The causes of death of four patients (16%) included primary graft dysfunction, bronchial dehiscence with long-term use of sirolimus, and uncontrollable infections. The median follow-up time from LT was 41.1 ± 25.0 months.

**Conclusions:** LT for LAM patients from the Asian population has been reinforced from the data that we presented. Peri-transplantation use of sirolimus and LAM-related complications should be further defined and under constant surveillance.

## Introduction

Lymphangioleiomyomatosis (LAM) is a rare disease characterized by the proliferation of abnormal smooth muscle-like cells (LAM cells), leading to diffuse pulmonary cyst formation, chylous pleural effusions, and the formation of lymphangioleiomyomas (1). LAM occurs sporadically (S-LAM) or as a pulmonary manifestation related to tuberous sclerosis complex (TSC-LAM) carrying mutations in TSC1 or TSC2 genes (2).

It generally affects women of reproductive age and presents a variable course, including indolent lung cysts, progressive dyspnea on exertion, recurrent pneumothorax, and thoracic lymphadenopathy, ultimately leading to respiratory failure, renal angiomyolipoma, and chylous effusions, as extrapulmonary manifestations. However, 30–40% of women with tuberous sclerosis complex developed pulmonary LAM (3). The inhibitors of the mechanistic target of rapamycin (mTOR) sirolimus have demonstrated treatment benefit in LAM (4–6).

When all medical therapies are exhausted, end-stage patients should be considered for lung transplantation (LT) (7), which is a definitive treatment option evidenced by increasing survival benefit and improvement in the quality of life (5, 8–17). The first LT was performed for LAM in 1984 (18). From 1987 to 2002, LAM accounted for only approximately 1% of all causes for LT, according to the data from the United Network for Organ Sharing (19). The survival rate seemed to be comparable with that of patients undergoing LT for other forms of end-stage lung diseases (1, 20). The results from the United States (19), Brazil (21), and France (12) have shown a preference for bilateral lung transplantation (BLT). More patients received BLT compared with single lung transplantation (SLT) between January 1995 and June 2014, according to the registry of the International Society for Heart and Lung Transplantation (ISHLT). However, a report from Japan chose SLT considering donor sharing; more than 80% of lung transplant recipients with LAM underwent SLT during 2000–2016 (22).

Moreover, limited published data are available regarding patients' clinical pretransplant status, types of procedure, posttransplant complications, and LAM-related morbidity in the current era. The primary aim of this study was to summarize the clinical data from patients with pulmonary LAM who underwent LT at centers in China, particularly documenting pretransplant features, posttransplant morbidity, and outcomes.

## Methods

### Study Cohorts and Data Collection

The institutional ethics committees of Wuxi People's Hospital and China–Japan Friendship Hospital approved the study, including the present retrospective review, verbal consent procedure, and analysis of data. All patient data were anonymous. Written informed consent was obtained from the patients or their next of kin. The study was conducted in accordance with the 2000 Declaration of Helsinki and the Declaration of Istanbul 2008. None of the transplant donors were from a vulnerable population, and all donors or next of kin provided written informed consent that was freely given.

Patients undergoing LT for end-stage pulmonary LAM from 2010 to 2018 were identified and reviewed. LAM diagnosis was based on the 2010 guidelines of the European Respiratory Society (1). All pretransplant diagnoses of pulmonary LAM were confirmed in the explants. All patients with LAM had undergone a systematic assessment. The first was to assess the ABO type, human leukocyte antigen type, and donor-specific human leukocyte antigen antibodies (DSA) of our patients. The second was to assess the function of target organs, involving lung function test, blood gas analysis, 6-min walk test (6MWT), and chest computed tomography (CT). The third concerned the function of other organs, including liver and kidney functions, coagulation function, bone marrow function, and heart function (electrocardiogram, echocardiography, and coronary CT angiography if necessary). The fourth was the exclusion of cancer, including blood cancer marker level, whole-body CT examination, and positron emission tomography-CT if necessary. The fifth related to whether LAM was associated with other organs, involving CT of the head, chest, and whole abdomen and B-ultrasound of the uterus and its accessories. The last concerned the preoperative infection status and the immunity level of patients. Preoperative discussions were conducted with multidisciplinary experts to determine whether the patient would be included in the waiting list for LT, including LT doctors, respiratory physicians, cardiologists, thoracic surgeons, anesthesiologists, emergency doctors, intensive care unit doctors, rehabilitation doctors, nutritionists, and ethics committee members. The surgical indications of patients with LAM at the center referred to the 2006 guidelines of ISHLT (23).

The ABO blood groups of the donors and recipients were identical before the surgery. The preoperative chest X-ray or chest CT examination did not find any pulmonary infection or other pulmonary diseases in the donors, with the oxygenation index reaching above 300 mmHg. Pretransplant demographics of recipients, clinical history, diagnostic methods to confirm LAM diagnosis, medical treatments, surgical characteristics, complications, morbidity, mortality, survival rate after LT, and use of sirolimus after transplantation were reviewed in all patients. The patients were followed up every 3 months during the first year after LT, 6 months after 1–3 years, and once annually after 3 years. The review covered the target organ and other organ functions and the immunity level and infection status of patients.

### Statistical Analysis

Descriptive statistics were used to analyze patient characteristics. Normally distributed continuous data were described as mean ± standard deviation. Categorical variables were presented as percentages. Medians and ranges were presented for skewed data. Survival was estimated by Kaplan–Meier analysis. All calculations and comparisons were performed using SPSS version 16 (SPSS Inc., IL, USA) and GraphPad Prism 7 (GraphPad Software Inc., CA, USA). A *P*-value of <0.05 was considered significant.

## Results

### Study Population and Establishment of Lymphangioleiomyomatosis Diagnosis

From January 2010 to December 2018, 25 female patients with sporadic LAM underwent sequential BLT at the centers. The main clinical characteristics before LT are described in Table 1. The mean ages at diagnosis and while undergoing LT were 35.0 ± 8.6 years and 36.8 ± 9.3 years, respectively.

**Table 1:** Clinical characteristics of LAM patients (*n* = 25) at registration for LT.

**Variable**	**Value**
Female (*n*,%)	25 (100)
Age at diagnosis (years)	35.0 ± 8.6
Age at transplantation (years)	36.8 ± 9.3
Pregnancy history (*n*, %)	19 (76)
**Clinical features**	
Patients with lung involvement alone (*n*, %)	15 (60)
Patients with extrapulmonary manifestation (*n*, %)	10 (40)
Renal angiomyolipoma (*n*, %)	6 (24)
Mediastinal lymphadenopathy (*n*, %)	2 (8)
Retroperitoneal lymphangioleiomyoma (*n*, %)	2 (8)
Pelvic lymphangioleiomyoma (*n*, %)	5 (20)
Pneumothorax (*n*, %)	21 (84)
Pleurodesis (*n*, %)	4 (16)
Chylous effusion (*n*, %)	3 (12)
Chylothorax (*n*, %)	3 (12)
Ascites (*n*, %)	1 (4)
Supplemental oxygen therapy (*n*, %)	25 (100)
Oxygenation index	168.1 ± 52.0
Use of sirolimus (*n*, %)	8 (32)
Use of sirolimus until lung transplantation (*n*, %)	8 (32)
Pulmonary function test (*n*, %)	6 (24)
FEV1 (%predicted)	15.9 ± 6.9
FVC (%predicted)	33.7 ± 19.6
6-min walk test	5 (20)
Distance (m)	85.4 ± 46.0
Minimum SpO2 (%)	81.8 ± 4.3

### Clinical and Radiologic Findings Before Transplantation

Twenty-one (84%) recipients had a history of pneumothorax, which was the most common pretransplant symptom, and four patients (16%) had been treated by pleurodesis. Ten patients (40%) presented with extrapulmonary manifestations of LAM (Table 1), involving the kidney, pelvic, mediastinal, and retroperitoneal regions. Three patients (12%) had chylothorax. The most common radiologic features on chest CT were diffuse, cystic lung disease consistent with LAM, further confirmed in the explants of all patients.

All the patients had severe hypoxia and required continuous oxygen before surgery; seven patients (28%) required mechanical ventilation before transplantation, and one patient (4%) received a tracheotomy. Due to the critical status, six (24%) patients completed the pulmonary function test, which showed FEV1 15.9 ± 6.9% of predicted and FVC 33.7 ± 19.6% of predicted. All patients used supplemental oxygen before transplant with a concentration of 29–53% and an oxygenation index of 168.1 ± 52.0. Five (20%) patients completed 6MWT. The mean distance walked was 85.4 ± 46 m, and the minimum SpO_2_ at the end of the test was 81.8 ± 4.32%. The last pulmonary function test and 6MWT results before LT are summarized in Table 1.

### Medical and Surgical Interventions Before Transplantation

Eight (32%) patients received sirolimus before transplantation, for an average time of 3.9 years (range 1–9 years). One patient repeatedly had a bilateral pneumothorax, atelectasis, intractable chylothorax, abdominal chylous fluid, and a uterine muscle lipoma. She received pleural and peritoneal drainage, followed by right middle lobe wedge resection with lung repair. A uterus lymphatic smooth muscle tumor was resected. Then, she received thoracic duct ligation and bilateral pleurodesis treatment. The postoperative histological examination confirmed lung and uterine LAM. Another four patients (16%) received pleurodesis due to recurrent pneumothorax and/or chylothorax. Moreover, 19 patients (76%) had a history of pregnancy, and 6 patients (24%) had a recent history of pregnancy (within 1 year of pregnancy, including abortion or production).

### Lung Transplantation and Posttransplantation Outcomes

No significant intraoperative complications were reported, although two patients (8%) required intraoperative extracorporeal membrane oxygenation support. The average volume of intraoperative blood loss was 1,280 ±730 ml, and the mean blood transfusion was 1,316 ± 874 ml. One patient (4%) experienced intraoperative blood loss of up to 4,000 ml due to extensive pleural adhesions in the thoracic cavity regarding pretransplant bilateral pleurodesis.

The complications after the surgery included the disturbance of anastomotic integrity. One patient developed anastomotic stenosis after anastomotic infection in the early stage after LT. The condition of anastomotic stenosis improved after antibiotics and balloon dilatation treatment. However, another patient died due to anastomotic leakage. After the surgery, 45% of the patients developed an infection, and two patients died of tuberculosis infection. One patient died of primary graft dysfunction during the early postoperative period. Bronchiolitis obliterans syndrome was diagnosed in one patient (4%) 48 months after LT. The postoperative details are described in Table 2. Twenty-one (84%) patients received triple-drug maintenance immunosuppressive therapy (tacrolimus, mycophenolate, and prednisone) after transplantation. Four patients (16%) temporarily received tacrolimus and prednisone treatment because of infection in an early stage after the surgery. One patient received sirolimus (maintained serum level 8–10 ng/ml) instead of tacrolimus 3 months after transplantation when anastomosis completely healed, but meanwhile, a retroperitoneal mass was suspected.

**Table 2:** Intra- and posttransplantation data (*n* = 25).

**Variable**	**Value**
Follow-up (months)	41.1 ± 25.0
**Immunosuppression**	
Tacrolimus + mycophenolate + prednisone (*n*, %)	21 (84)
Tacrolimus + prednisone (*n*, %)	4 (16)
**Complications**	
Neoplasm or lymphoproliferative disease (*n*, %)	0 (0)
Chylothorax (*n*, %)	1 (4)
Recurrence of LAM (*n*, %)	0 (0)
Pulmonary retransplantation (*n*, %)	0 (0)
Intraoperative bleeding (*n*, %)	1 (4)
Anastomotic complications (*n*, %)	2 (8)
Anastomotic leakage (*n*, %)	1 (4)
Anastomotic stenosis (*n*, %)	1 (4)
Bronchiolitis obliterans syndrome (*n*, %)	1 (4)
Recurrence of LAM (*n*, %)	0 (0)
Primary graft dysfunction (*n*, %)	1 (4)
Infection (*n*, %)	12 (48)
Tuberculosis infection	3 (12)
Aspergillus	3 (12)
Acinetobacter baumannii	4 (16)
Other infection	2 (8)
**Cause of death**	
Primary graft dysfunction (*n*, %)	1 (4)
Anastomotic leakage (*n*, %)	1 (4)
Infection (*n*, %)	2 (8)

The median follow-up of this group of patients was 41.1 ± 25.0 months. The range of follow-up from LT to either death or closing date was 0.5–84 months. Four deaths (16%) occurred during the study. One patient (4%) died of primary graft dysfunction 30 days after the surgery. Two patients died of uncontrollable infections 2 and 13 months after the surgery. Another patient (4%) died of anastomotic leakage 12 days after the surgery. The patient had been on oral sirolimus for 9 years and until transplantation.

**Figure 1:**
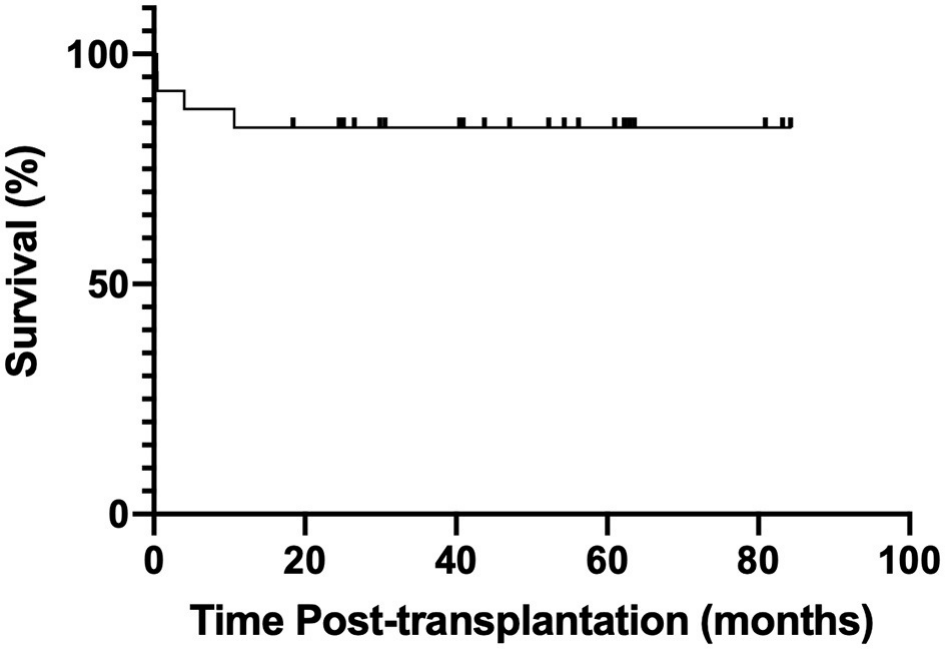
Kaplan–Meier survival analysis after lung transplantation in 25 patients with LAM.

No LAM recurrence was observed, no patient developed the malignant proliferative disease after the procedure, and no patient underwent pulmonary re-transplantation. The estimated 1- and 2-year posttransplant survival rates were 88 and 84%, respectively (Figure 1).

## Discussion

This study demonstrated a favorable outcome for LT in LAM patients similar to that published in a USA report from actuarial Kaplan–Meier survival for LAM was 85% at 1 year, and 76% at 3 years (19). Despite three (12%) early deaths (<3 months after transplantation), the survival rate of the present cohort was more favorable than that of patients with other indications (24) and Japan's data, as well as the 1-year survival of 78–81% in the report of ISHLT registry (25). The estimated survival rates from a Brazilian cohort have been shown to be 90% after 1 year, 90% after 3 years, and 77% after 5 years, which were greater than those observed in previous studies (15–20). The long-term survival was also promising, attributing to the fact that it often involved younger patients without any extrapulmonary organ dysfunction (11).

A newly adopted lung allocation scoring system based on disease severity in the USA since 2005 (26) has categorized LAM with chronic obstructive pulmonary disease and other obstructive diseases, giving a lower priority to patients with idiopathic pulmonary fibrosis when allocating donor lungs. Thus, a longer waiting time for patients with LAM should be optimized. Recently, BLT has been performed with a higher frequency compared with SLT for LAM (13, 15, 27). BLT could achieve a better long-term outcome (28) due to lower incidence of bronchiolitis obliterans syndrome, reducing the rate of complications such as pneumothorax, chylothorax, and recurrence of LAM following LT, together with better pulmonary function tests. Hence, BLT is recommended if donor lungs are available.

Recurrent pneumothorax and chylothorax requiring surgical or chemical pleurodesis are common in patients with LAM. The average lifetime pneumothorax-related burden for patients with LAM is a high in-patient cost (29). The upmost challenge for LT in patients with LAM would be to minimize the risk of severe hemorrhage in patients who had pretransplant pleurodesis for pneumothorax or chylothorax, which is a serious triggering factor for excessive bleeding by dissection of the adhesions. According to the previous reports, the pretransplant rates of pleurodesis and pleurectomy ranged from 18 to 100% and from 21 to 43%, respectively, whereas the rate of intraoperative complications varied from 25 to 71% (11–17) (Table 3). However, in the present cohort with a pleurodesis rate of 16%, the rate of intraoperative complications was only 4%.

**Table 3:** Summaries of studies on LT for LAM.

**Study**	**No. of LT**	**Age at LT (years)**	**FEV1%**	**FEV1/FVC**	**Pre-LT complications**	**Intra-surgery**	**Main Post-LT complications**	**30 days of mortality**	**1-year survival**
Boehler et al. (9)	34	40 ± 9	24 ± 12	38 ± 12	Pleural effusion 16% Pleurodesis 18%	Adhesions 54% Hemorrhage 12%	Bronchial stenosis or dehiscence 18%	15%	69%
Urban et al. (10)	13	-	FEV1 0.57 ± 0.15 L	-	Pleurodesis 58% (in total)	-	-	8%	69%
Pechet et al. (11)	14	41.8 ± 6.8	20 ± 8	-	Pleural effusion 7.1% Pleurodesis 00%	Adhesions 71.4% Hemorrhage 50%	Sepsis 21.4% Dehiscence 14.3% Chylothorax 28.6%	0%	100%
KpodonuJ et al. (19)	79	41.1 ± 8.3	-	-	-	-	Fungal infection 2.5%	5%	86%
Reynaud-Gauber et al. (12)	45	41 ± 10	Obstructive 26.4 ± 14.1 Combined19.7 ± 7.2	Combined 47.4 ± 12.3	Pleural effusion 13.3% Pleurodesis 48.8%	Adhesions 46.6% Hemorrhage 46.6%	Bronchial stenosis or dehiscence 15.6%	-	80%
Benden et al. (13)	61	41.3 ± 9.1	27 ± 14	-	Pleural effusion 14% Pleurodesis 54.1%	Adhesions 57.4% Hemorrhage 33.3%	Respiratory tract infection 59%	5%	79%
Machuca et al. (14)	10	43.8 ± 6.7	Mean 32.9	-	Pleural effusion 0% Pleurodesis 100%	Hemorrhage (>1,000 ml) 30%	Infections 30%	10%	90%
Nakagirin et al. (16)	13	42 ± 4.6	-	-	Pleural effusion 30.7% Pleurodesis 53.8%	-	-	-	-
Ando et al. (17)	57	39.5 ± 7.3	32.8 ± 17	40.2 ± 15.2	Pleural effusion 21% Pleurodesis 53.8%	-	Bronchial stenosis or dehiscence 0%		93.5%
Bruno et al. (21)	11	43 ± 7	28 ± 14	45 ± 16	Pleurodesis 9%	-	Stenosis 9% Pulmonary infections 55%		90%
Oishi et al. (22)	29	45.3 ± 8.4	-	-	Pleural effusion 6.9% Pleurodesis 31%	Adhesions 31% Hemorrhage (>2,000 ml) 13.8%	Chylothorax 20.7%	0%	83.3%
Khawar et al. (30)	138	45 (38–52)	23 (17.0–33.3)	-	-	-	-	-	94%
Salman et al. (31)	25	50 ± 9	22 ± 10	-	-	-	Dialysis (32%)	0%	92%
Kurosaki et al. (32)	12	32 ± 7.3	FEV1 0.7 ± 0.4		Pleurodesis (58%)		Chylothorax 50% Acute rejection 42%	0	100%

Previous studies showed that almost all patients with LAM died 10 years after the start of symptoms (33, 34). The guideline for LAM recipients from ISHLT suggested that the New York Heart Association functional class III or IV, severe impairment of lung function, and poor exercise tolerance should be considered for LT (23). When determined to perform LT, severe obstruction, compromised gas exchange impairing quality of life, and progressive respiratory failure are among the most important factors for evaluation (35). How to refine the specific timing of LT is very difficult. The poor prognostic factors included decreased FEV_1_/FVC, decreased total lung capacity, and cystic lesions. In the published reports of cohorts, the age at onset, diagnosis, diffusing capacity for carbon monoxide (DLCO)% predicted, PaO_2_, and 6MWT at registration were quite similar. However, FEV_1_% predicted at registration and during the waiting period have been shown to be worthy of special note. In the present cohort, most of the patients in the present study had end-stage respiratory failure and required oxygen or even high-flow oxygen to extend their life before the surgery. Most of them could not complete the pulmonary function test. As few as six patients could complete the necessary ventilatory function test as required but were unable to undergo the diffusion function test, as well as 6MWT. The pretransplant FEV_1_% was 15.9 ± 6.9% of predicted and obviously lower than that found in most previous reports, ranging from 19 ± 11% to 32.8 ± 17% (11–17). This signified the difficulties related to patient management at the centers to perform BLT. Although pleural intervention is not seen as a contraindication to transplant, it would become a contributor to both intra-surgery and post-surgery morbidities.

In the present cohort, the waiting time of patients seemed relatively shorter than the Japanese center's interval, partly due to the donation volume and the endurance of the patients (17). The time to choose LT for patients with LAM was also crucial for long-term survival. With the delay in choosing LT, intractable chylothorax or metastatic LAM lesions could be seen. If patients received LT in a later phase, critical status, high cost, and risk of retransplant were implicated. More assessment tools are needed in the future to fully evaluate the timing and benefit of LT in patients with LAM.

Infections are a major complication after LT in LAM. The frequency of respiratory infections ranged from 10 to 59%, with heterogeneous etiology such as cytomegalovirus, *Aspergillus* sp., and bacterial pathogens (11–16). Two patients died of uncontrollable infections pathologically confirmed as tuberculosis infection. Another surviving patient also had a relapsed tuberculosis infection 6 months after the surgery. This patient recovered well after antituberculosis treatment. Hence, patients with LAM undergoing LT are recommended to receive conventional inhalation isoniazid to prevent tuberculosis infection. Immunosuppression use after LT is also a risk factor.

For patients with LAM, the use of mTOR inhibitors is a topic with special interest. As a conventional immunosuppressant in organ transplantation (36), sirolimus has been proved *in vitro* and in preclinical models for stabilizing lung function in LAM and treating extrapulmonary manifestations, such as renal angiomyolipoma, by inhibiting T- and B-lymphocyte activation *via* the IL-2 signaling pathway (3, 37). mTOR inhibitors are appealing to physicians in decreasing the rate of cytomegalovirus infection (38) and effective for post-surgery chylous pleural and peritoneal effusions (3). A significant concern regarding the use of mTOR inhibitors is that these drugs interfere with wound healing corresponding to the suppression of fibroblast proliferation and angiogenesis, which is related to the increased rate of bronchial anastomotic integrity disorder (39). In the present cohort, one patient died of anastomotic leakage 12 days after the surgery. She had received sirolimus for 9 years before the surgery and did not stop treatment before the surgery. No anastomotic leakage was observed in the other seven patients treated with sirolimus medication for 1–6 years. Whether it is necessary to stop the drug before the surgery and when need to be confirmed by further exploration. Some centers suggest initiating mTOR inhibitors only after confirming complete bronchial anastomotic healing, 3 months (12) after LT or even waiting up to 9 months. A high incidence of airway dehiscence occurred when sirolimus was begun immediately after transplantation (40).

Previous studies found the predominance of LAM in women of reproductive age. Pregnancy worsened the disease (41), and menopause caused disease remission (42). Patients enrolled in the present cohort seemed to be younger and mainly sporadic LAM dominant compared with clinical analysis findings from the Chinese mainland (43) and the American National Heart, Lung and Blood Institute (2, 35). Exploring whether the younger age of female patients to undergo LT can improve their life expectations, including pregnancy demanding, can be highly challenging.

This study had certain limitations because of the paucity of available knowledge regarding the pathophysiology and treatment of LAM. Small sample size and its retrospective heterogeneous characteristic were the main factors to be considered as insufficiencies similar to previous reports due to the rarity of LAM and a high requirement of the large-center qualification. The condition of patients was quite serious in the present study, and most patients could not complete the preoperative lung function and 6MWT, resulting in incomplete data. Hence, large-scale clinical research is needed in the future. For the surveillance of the posttransplantation course, convincing evidence of maintenance therapeutic agents and improvements in patients with LAM undergoing transplantation with regard to their posttransplant survival will be the key points to explore in future studies.

## Conclusions

The present study reinforced the role of LT for patients with end-stage LAM. Favorable survival and quality of life were demonstrated. Nevertheless, the occurrence of LAM-related and transplant-related complications should be monitored continuously. Pre- and posttransplantation immunosuppressive approaches confer a special interest in further demystifying LAM *via* coordination of medical therapy providers and transplant surgeons, thus allowing for the optimization of recipients both while waiting and after the surgery.

## Data Availability Statement

The original contributions presented in the study are included in the article/supplementary material, further inquiries can be directed to the corresponding author/s.

## Ethics Statement

The studies involving human participants were reviewed and approved by The Institutional Ethics Committees of Wuxi People's Hospital The Institutional Ethics Committees of China-Japan Friendship Hospital. The patients/participants provided their written informed consent to participate in this study.

## Author Contributions

JZ and JC take full responsibility for the content of this manuscript, including its data and analysis. DL, BY, LB, MZ, HW, JL, BW, and K-FX made substantial contributions to the conception and design of the study. JZ and WC made substantial contributions to the analysis and interpretation of data. JZ drafted the initial manuscript. All authors read and approved the final manuscript.

## Conflict of Interest

The authors declare that the research was conducted in the absence of any commercial or financial relationships that could be construed as a potential conflict of interest.
